# Improving genetic diagnostic yield in a large cohort of children with rare vascular anomalies or *PIK3CA*-related overgrowth spectrum

**DOI:** 10.1016/j.gimo.2023.100837

**Published:** 2023-10-17

**Authors:** Timothy E. Green, Denisse Garza, Natasha J. Brown, Michelle G. de Silva, Mark F. Bennett, Caitlin Tubb, Roderic J. Phillips, Duncan MacGregor, Susan J. Robertson, Phillip Bekhor, Jodie Simpson, Anthony J. Penington, Michael S. Hildebrand

**Affiliations:** 1 Epilepsy Research Centre, Department of Medicine (Austin Hospital), University of Melbourne, Heidelberg, Australia; 2 Victorian Clinical Genetics Services, Melbourne, Parkville, Australia; 3 Tasmanian Clinical Genetics Service, Hobart, Tasmania, Australia; 4 Murdoch Children’s Research Institute, Melbourne, Parkville, Australia; 5 Royal Children’s Hospital Department of Paediatrics, University of Melbourne, Parkville, Australia; 6 Population Health and Immunity Division, The Walter and Eliza Hall Institute of Medical Research, Melbourne, Australia; 7 Department of Medical Biology, University of Melbourne, Parkville, Australia; 8 Department of Plastic and Maxillofacial Surgery, Royal Children’s Hospital, Melbourne, Australia; 9 Department of General Medicine, Royal Children’s Hospital, Melbourne, Australia; 10 Department of Paediatrics, Monash University, Clayton, Australia; 11 Anatomical Pathology, Royal Children's Hospital, Parkville, Australia; 12 Department of Dermatology, Royal Children’s Hospital, Melbourne Australia; 13 Facial Sciences Research Group, Murdoch Children’s Research Institute, Parkville, Australia

**Keywords:** Droplet digital PCR, Genetic diagnosis, Mosaicism, PROS, Vascular anomalies

## Abstract

**Purpose:**

Drugs that attenuate hyperactivation of the phosphatidylinositol 3-kinase-Akt and Ras-mitogen-activated protein kinase signaling pathways are emerging treatments for children with rare, intractable vascular anomalies or *PIK3CA-*related overgrowth spectrum (PROS) with an eligible genetic diagnosis. However, access to genetic testing remains a barrier to genetic diagnosis. Here, we implement a targeted molecular diagnostic strategy for vascular anomalies or PROS.

**Methods:**

We applied a novel genetic testing strategy to children with vascular anomalies or PROS using a tiered approach of (1) droplet digital PCR, (2) Sanger sequencing, (3) high-depth exome sequencing, and (4) reanalysis of existing clinical exome data.

**Results:**

We applied this strategy to 60 individuals detecting pathogenic somatic variants in 33 of 60 (55%). This included 26 individuals with slow-flow lesions with variants in *PIK3CA*, *TEK*, *GNAQ*, *GNA11, BRAF*, or *PIK3R1*, 4 individuals with fast-flow lesions with variants in *KRAS* or *MAP2K1*, 1 individual with a *PIK3CA* variant and a mixed phenotype, and 2 individuals with *PIK3CA* variants and PROS without vascular anomalies.

**Conclusion:**

We demonstrate an effective genetic diagnostic strategy for children with vascular anomalies or PROS identifying somatic variants in 55% of individuals. Increasing genetic diagnostic yield extends the clinicogenetic spectrum and may provide access for those with intractable disease to therapeutic drug trials.

## Introduction

**Figure 1:**
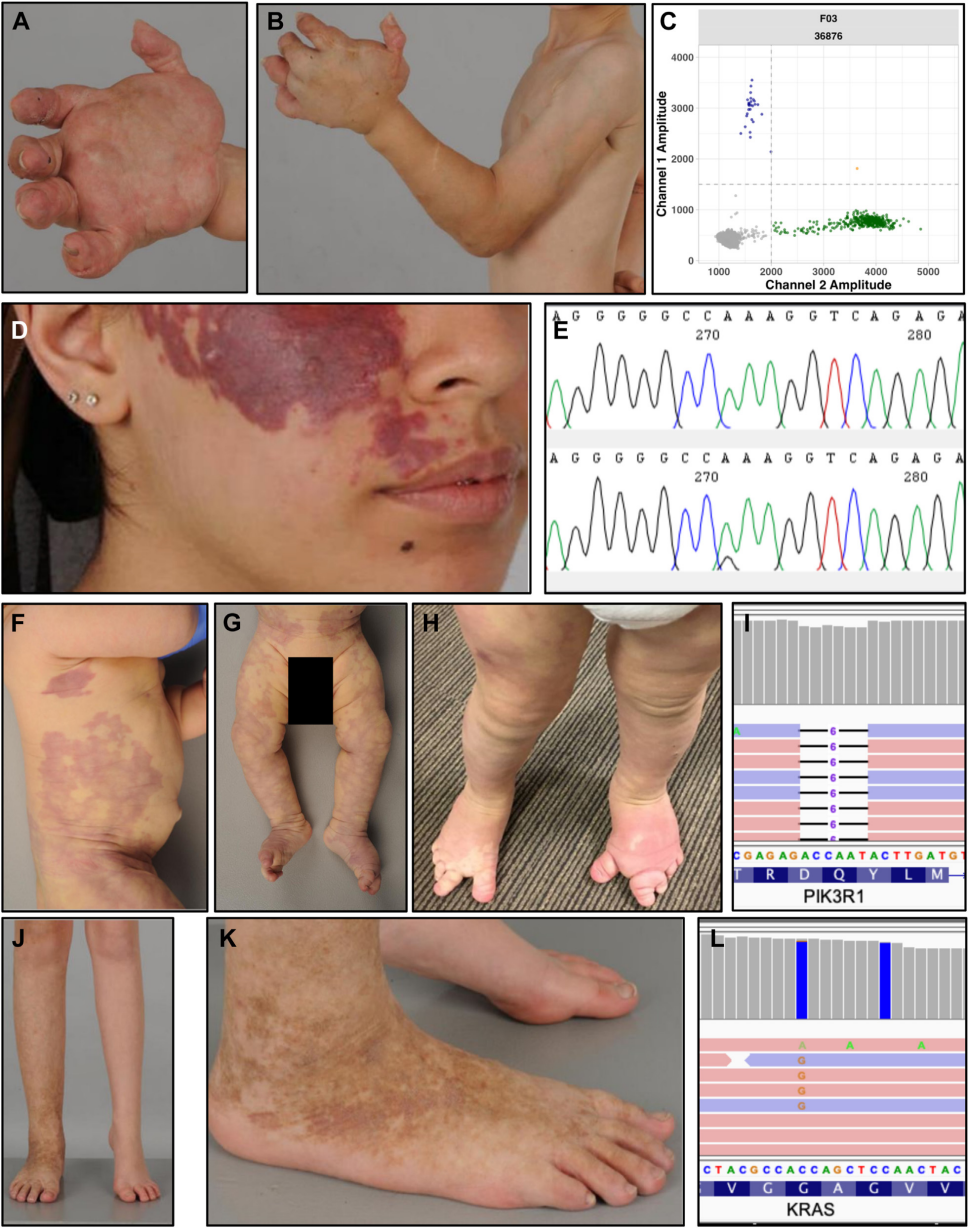
**Clinical photographs and molecular analyses.** A-B. Case 9 demonstrating extensive overgrowth of left hand and upper limb with associated mixed lymphatic and venous malformation. C. Droplet digital PCR (ddPCR) detection of the *PIK3CA* (NM_006218.4):c.1633G>A p.(Glu545Lys) variant in the VM tissue of case 9 at a VAF of 6.17%. Variant DNA copies (blue droplets), wild-type DNA copies (green droplets), droplets without DNA copies (gray droplets), droplets with multiple DNA copies (orange droplets). The *x*-axis shows the amplitude of wild-type fluorescent probe; the *y*-axis shows the amplitude of variant specific fluorescent probe. D. Case 26 presented with a facial port wine stain with nodularity, including pyogenic granuloma. E. Sanger sequencing of case 26 demonstrating the detection of the *GNAQ* (NM_002072.5):c.626A>G p.(Gln209Arg) variant in the CM tissue (bottom electropherogram). The variant was not detected in blood derived DNA (top electropherogram). F-G. Case 33 at age 3 months demonstrating extensive reticulate CM involving the trunk, abdomen, groin, legs, and both feet and overgrowth of left leg and foot. H. Case 33 at age 3 years demonstrating extensive bilateral CM, macrodactyly, syndactyly, relative overgrowth of right foot and leg, and bilateral sandal gap. I. The Integrative Genomics Viewer screenshot shows the *PIK3R1* (NM_181523.3):c.1735_1740del p.(Gln579_Tyr580del) in case 33 in 15/220 reads by high-depth research exome sequencing. Pink and blue colors indicate sequencing reads on the forward and reverse strands, respectively. J-K. Images of case 22 show overgrowth of the right leg and foot with mild hemosiderin staining of distal right leg and foot with an underlying aggressive arteriovenous malformation. L. The Integrative Genomics Viewer screenshot shows the reanalysis of existing clinical ES data for case 22, which revealed the *KRAS* (NM_004985.5):c.35G>C p.(Gly12Ala) variant in 4 of 159 reads. Pink and blue colors indicate sequencing reads on the forward and reverse strands, respectively.

Vascular anomalies arise through abnormal blood or lymphatic vessel development and can present with a wide range of health problems, from simple cutaneous lesions to severe life-threatening complex disease (Figure 1). Vascular anomalies may be progressive and associated with significant complications including bleeding risk, thrombosis, pulmonary emboli, cerebral artery dysplasia, renal anomalies, epidermal naevi, soft-tissue tumors, focal overgrowth, scoliosis, and major disfigurement.^1^ Although a minority of children with vascular anomalies inherit a germline pathogenic variant, most are sporadic, caused by post-zygotic (mosaic) somatic variants with low-variant allele fraction (VAF) and variable distribution within lesional tissue.^2^ Similarly, *PIK3CA*-related overgrowth spectrum (PROS) caused by somatic variants in *PIK3CA*, is a heterogenous multi-system disorder delineated by overgrowth with additional features, which may include vascular anomalies.^3^

To date, most of the somatic variants identified in vascular anomalies or PROS occur within genes of 2 major developmental signaling cascades—the Ras-mitogen-activated protein kinase (MAPK) and phosphatidylinositol 3-kinase (PI3K)-Akt pathways.^2^ These 2 pathways control the proliferation and differentiation of several cell types, including vascular endothelial cells.^2^ Robust genotype-phenotype correlations have now been established, with variants in the PI3K-Akt pathway generally associated with abnormal development of capillaries, veins, or lymphatics (collectively “slow-flow” anomalies), whereas variants in the Ras-MAPK pathway are typically associated with abnormal development of arterial vessels (collectively “fast-flow” anomalies).^2^ Nonetheless, there are exceptions to these distinct associations, such as post-zygotic variants in Ras-MAPK genes identified within slow-flow malformations, including complex lymphatic anomalies, highlighting the broad clinico-genetic spectrum of vascular anomalies.^4^

Obtaining a genetic diagnosis for vascular anomalies or PROS can be challenging. Routine clinical genetic testing involves interrogation of germline or *de novo* variation by analyzing peripheral DNA samples. This does not permit the detection of somatic variants restricted to lesional tissue. Thus, genetic diagnosis depends on access to small lesional biopsies that can be distressing or painful for children and can be challenging to perform because of bleeding risk from vascular lesions. There are also significant challenges to the genetic testing. First, clinical genetic testing for vascular anomalies is not universally funded by hospitals or health care providers. Second, causative variants are typically at low VAF (eg, <10%) in lesional tissue, meaning that sensitive detection methods are required, which may not be available in all clinical testing laboratories.^5^

Here, we describe a sequential tiered genetic testing strategy that leverages the well-characterized genetic architecture of these disorders to provide a rapid and cost-effective approach to genetic diagnosis.

## Materials and Methods

### Cohort and samples

Individuals were recruited from a specialized Vascular Anomaly Clinic. Genomic DNA was extracted from either fresh frozen tissue samples up to 30 mg or archival formalin-fixed paraffin-embedded (FFPE) tissue samples up to 10 × 10 μM shavings using the Qiagen All Prep DNA/RNA or QIAamp DNA FFPE Tissue Kit. If available, peripheral DNA was extracted from saliva or whole blood using the DNA Genotek prepIT.L2P and the Qiagen QIAamp DNA Blood Maxi Kit according to the manufacturer’s instructions.

### Droplet digital PCR and Sanger sequencing

TaqMan SNP Genotyping Assays from Thermo Fisher Scientific Inc were used to detect and quantify 29 selected recurrent variants (Supplemental Table 1). Droplet generation, PCR cycling, and droplet reading were performed according to the manufacturer’s recommendations (Bio-Rad). Sanger sequencing was performed using gene-specific primers with the BigDye v3.1 Terminator Cycle Sequencing Kit (Applied Biosystems) and 3730 XL DNA Analyzer (Applied Biosystems) (Supplemental Table 1).

### Exome sequencing analysis

If available, existing non diagnostic clinical exome data were obtained for reanalysis on a research basis from the clinical laboratory. For those individuals who underwent research exome sequencing (ES), DNA derived from affected tissue was sequenced with the Agilent SureSelect DNA Human All Exon V6, 96RXN kit (Agilent Technologies) and the Illumina NovaSeq 6000 System (Illumina) with 150 bp paired-end reads targeting 400X coverage. For analysis, reads were aligned to the hg38 reference genome with BWA-MEM v0.7.17-r1188, then duplicate marking and base quality score recalibration performed with the Genome Analysis Toolkit (GATK).^6^^,^^7^ Germline variant calling was performed with GATK HaplotypeCaller and somatic variant calling with GATK Mutect2 v4.0.1.2.^6^ Variants were annotated using vcfanno and ANNOVAR.^8^^,^^9^

We considered variants only within a curated list of 68 genes associated with vascular anomalies (Supplemental Table 2). Variants were filtered accordingly: located in a coding or splice site region, frequency <0.001 in the Genome Aggregation Database (gnomAD v2.1.1), and variant type—missense, nonsense, coding indel, or splice site.^10^

## Results

We studied a cohort of 60 individuals with vascular anomalies or PROS consisting of 44 individuals with slow-flow, 11 with fast-flow, 3 with mixed anomalies, and 2 with PROS without vascular anomalies. We initially performed droplet digital PCR (ddPCR), a highly sensitive technique designed to detect low-level mosaicism, on tissue-derived DNA samples. We selected 29 recurrent variants frequently identified in vascular anomalies or PROS to target by ddPCR (Supplemental Table 1). Next, targeted Sanger sequencing of hotspot regions of genes associated with vascular anomalies was conducted to detect variants beyond those most frequently reported (Supplemental Table 1). Lastly, research ES was completed on lesional tissue on a subset of individuals (*n* = 13) who remained unsolved. In 5 cases that clinical ES had previously been non diagnostic, we performed reanalysis to identify variants missed by clinical analytic pipelines.

We identified pathogenic somatic variants in 33 of 60 (55%) individuals, by ddPCR, Sanger sequencing, research ES, and clinical ES reanalysis (Table 1, Figure 1).

**Table 1:** Genetic findings in 33 individuals with vascular anomalies or PROS

#	ID	Tissue Tested	Phenotype	Gene	Variant	HGVS GRCh38	Method	VAF Lesion	VAF Other
*1*	*36114*	VM/LM	PROS	*PIK3CA*	c.1636C>A p.(Gln546Lys)	NC_000003.12:g.179218306C>A	Sanger	NT	NT
*2*	*36154*	CM/LM	PROS	*PIK3CA*	c.1633G>A p.(Glu545Lys)	NC_000003.12:g.179218303G>A	ddPCR	18.80%	0%^Blood^
*3*	*36333*	VM	AVM brain, VM hand	*PIK3CA*	c.3140A>G p.(His1047Arg)	NC_000003.12:g.179234297A>G	ddPCR	1.67%	0%^Saliva^
*4*	*36657*	VM	Multifocal VM	*PIK3CA*	c.1633G>A p.(Glu545Lys)	NC_000003.12:g.179218303G>A	ddPCR	2.66-11.7%	0%^Blood^
*5*	*36801*	LM	LM head/neck	*PIK3CA*	c.1624G>A p.(Glu542Lys)	NC_000003.12:g.179218294G>A	ddPCR	4.89-7.18%	NT
*6*	*36155*	LM	LM head/neck	*PIK3CA*	c.1633G>A p.(Glu545Lys)	NC_000003.12:g.179218303G>A	ddPCR	1.89%	NT
*7*	*36407*	LM	LM chest	*PIK3CA*	c.1633G>A p.(Glu545Lys)	NC_000003.12:g.179218303G>A	ddPCR	1.00-8.40%	NT
*8*	*36717*	LM	LM head/neck	*PIK3CA*	c.3140A>G p.(His1047Arg)	NC_000003.12:g.179234297A>G	ddPCR	3.51%	0%^Blood^
*9*	*36876*	VM	PROS	*PIK3CA*	c.1633G>A p.(Glu545Lys)	NC_000003.12:g.179218303G>A	ddPCR	6.17%	NT
*10*	*36642*	LM	LM head/neck	*PIK3CA*	c.3140A>G p.(His1047Arg)	NC_000003.12:g.179234297A>G	ddPCR	4.54-8.58%	NT
*11*	*37985*	LM	LM left buttock	*PIK3CA*	c.1633G>A p.(Glu545Lys)	NC_000003.12:g.179218303G>A	ddPCR	3.56%	NT
*12*	*38053*	LM	LM tongue	*PIK3CA*	c.1633G>A p.(Glu545Lys)	NC_000003.12:g.179218303G>A	ddPCR	1.47%	0%^Saliva^
*13*	*36854*	CM	CM leg	*PIK3CA*	c.1093G>A p.(Glu365Lys)	NC_000003.12:g.179204536G>A	ES^R^	6.39-6.74%	NT
*14*	*37528*	LM	LM head/neck	*PIK3CA*	c.1624G>A p.(Glu542Lys)	NC_000003.12:g.179218294G>A	ddPCR	4.83%	NT
*15*	*37106*	Lipomatosis	FIL	*PIK3CA*	c.3140A>G p.(His1047Arg)	NC_000003.12:g.179234297A>G	ddPCR	3.93-9.05%	NT
*16*	*38157*	Affected skin	FO, macrodactyly with syndactyly	*PIK3CA*	c.1624G>A p.(Glu542Lys)	NC_000003.12:g.179218294G>A	ddPCR	24.80%	NT
*17*	*36120*	VM	Diffuse VM	*TEK*	c.2740C>T p.(Leu914Phe)	NC_000009.12:g.27212760C>T	ES^C^	6.40-20.5%	0.15%^Blood^
*18*	*36156*	VM	Multifocal VM	*TEK*	c.2740C>T p.(Leu914Phe)	NC_000009.12:g.27212760C>T	ddPCR	0.80%	NT
*19*	*36798*	VM	VM leg	*TEK*	c.2740C>T p.(Leu914Phe)	NC_000009.12:g.27212760C>T	ddPCR	4.05%	NT
*20*	*36129*	VM	Multifocal VM	*TEK*	c.2690A>G p.(Tyr897Cys)	NC_000009.12:g.27212710A>G	ddPCR	0.72%	NT
*21*	*36295*	VM	VM	*TEK*	c.2740C>T p.(Leu914Phe)	NC_000009.12:g.27212760C>T	ddPCR	3.42%	0%^Blood^
*22*	*36329*	Skin adjacent to AVM	AVM leg	*KRAS*	c.35G>C p.(Gly12Ala)	NC_000012.12:g.25245350C>G	ES^C^	6.84%	0%^Blood^
*23*	*38134*	AVM	AVM hand	*KRAS*	c.34G>T p.(Gly12Cys)	NC_000012.12:g.25245351C>A	ddPCR	1.27%	0%^Saliva^
*24*	*36499*	AVM	AVM hand	*MAP2K1*	c.171G>T p.(Lys57Asn)	NC_000015.10:g.66435117G>T	ddPCR	9.98%	NT
*25*	*38235*	AVM	NF1 + AVM lip	*MAP2K1*	c.171G>T p.(Lys57Asn)	NC_000015.10:g.66435117G>T	ddPCR	2.62%	NT
*26*	*36119*	CM	PWS + PG face	*GNAQ*	c.626A>G p.(Gln209Arg)	NC_000009.12:g.77794572T>C	Sanger	NT	NT
*27*	*38324*	CM	CM + OG	*GNAQ*	c.548G>A p.(Arg183Gln)	NC_000009.12:g.77797577C>T	ddPCR	3.39%	NT
*28*	*36875*	CM/VM/LM	KTS	*PIK3CA*	c.328_330del p.(Glu110del)	NC_000003.12:g.179199153_179199155del	ES^R^	10.5-12.4%	NT
		CM/VM/LM	KTS	*GNAQ*	c.626A>C p.(Gln209Pro)	NC_000009.12:g.77794572T>G		6.55-9.67%	NT
*29*	*36324*	PG + CM	CM + PG, EN	*GNAQ*	c.548G>A p.(Arg183Gln)	NC_000009.12:g.77797577C>T	ddPCR	6.34-15.8%	NT
		EN	CM + PG, EN	*PIK3CA*	c.1633G>A p.(Glu545Lys)	NC_000003.12:g.179218303G>A		18.80%	NT
*30*	*37527*	CM + PG	PWS + PG face	*GNAQ*	c.548G>A p.(Arg183Gln)	NC_000009.12:g.77797577C>T	ddPCR	1.43-12.30%	NT
		PG	PWS + PG face	*BRAF*	c.1799T>A p.(Val600Glu)	NC_000007.14:g.140753336A>T		3.40-18.42%	NT
*31*	*36334*	CM/VM	CMTC back	*GNA11*	c.627G>T p.(Gln209His)	NC_000019.10:g.3118945G>T	ES^R^	8.46%	NT
*32*	*38113*	CM	CM	*GNA11*	c.627G>T p.(Gln209His)	NC_000019.10:g.3118945G>T	ddPCR	9.27%	NT
*33*	*36310*	CM	PROS	*PIK3R1*	c.1735_1740del p.(Gln579_Tyr580del)	NC_000005.10:g.68295314_68295319del	ES^R^	6.05%	1.73%^Blood^; 1.93%^Saliva^

*AVM,* arteriovenous malformation; *CM*, capillary malformation; *CMTC*, cutis marmorata telangiectatica congenita; *ddPCR*, droplet digital PCR; *EN*, epidermal naevus; *ES*, exome sequencing; *ES^C^*, clinical exome sequencing reanalysis; *ES^R^*, high depth research exome sequencing; *FIL*, facial infiltrating lipomatosis; *FO*, focal overgrowth; *KTS*, Klippel Trenaunay Syndrome; *LM*, lymphatic malformation; *NF1*, neurofibromatosis type I; *NT*, not tested; *OG*, overgrowth; *PG*, pyogenic granuloma; *PROS*, PIK3CA-related overgrowth spectrum; *PWS*, port-wine stain; *VAF*, variant allele fraction as tested by ddPCR; *VM*, venous malformation.

RefSeq: *PIK3CA*: NM_006218.4, *TEK*: NM_000459.5, *KRAS*: NM_004985.5, *MAP2K1*: NM_002755.4, *GNAQ*: NM_002072.5, *BRAF*: NM_004333.6, *GNA11*: NM_002067.5, *PIK3R1*: NM_181523.3

We identified somatic variants within 26 of 44 (59%) individuals with slow-flow anomalies (either lymphatic malformation, venous malformation (VM), or mixed lymphatic/venous malformations) within the genes *PIK3CA*, *TEK*, *GNAQ*, *BRAF*, *GNA11*, and *PIK3R1* with VAFs ranging between 0.7% to 24.8% (Table 1). Three patients with slow-flow lesions had 2 somatic variants identified with a *GNAQ* variant in combination with either a *PIK3CA* or *BRAF* variant (Table 1).

Within 4 of 11 (36%) individuals with fast-flow lesions (arteriovenous malformations [AVM]) we identified somatic variants in *KRAS* and *MAP2K1* with VAFs ranging from 1.27% to 9.98% (Table 1). We identified a somatic *PIK3CA* variant in 1 individual with a mixed phenotype of AVM and VM at a VAF of 1.67% in the VM tissue (Table 1, case 3).

In 2 individuals with PROS without vascular anomalies, we identified somatic variants in *PIK3CA* with VAFs ranging 3.93% to 24.80% (Table 1, cases 15 and 16).

## Discussion

Genetic testing is increasingly important in the diagnosis of vascular anomalies or PROS with clinical trials or off-label use of new or repurposed drugs targeting hyperactivated signaling pathways improving outcomes in some individuals with previously intractable disorders.^11^, ^12^, ^13^, ^14^, ^15^ A genetic diagnosis may provide eligibility for clinical trials or inform off-label therapeutic interventions; however, identifying pathogenic somatic variants, often with low VAF, is challenging and the technologies required are not widely available.

Our diagnostic yield was achieved by applying multiple complimentary analysis strategies, including ddPCR, Sanger sequencing, research-based deep ES on tissue-derived DNA, and clinical ES reanalysis. All methodologies delivered diagnostic utility with variants identified in 25 of 59 (42%) by ddPCR, 2 of 15 (13%) by hot spot Sanger sequencing, 4 of 13 (31%) by deep tissue based ES, and 2 of 5 (40%) by clinical exome reanalysis (Table 1).

Overall, our strategy achieved a relatively high diagnostic yield of 55%, slightly below some published reports of similarly large cohorts of 41%,^5^ 63%,^16^ and 69%,^17^ albeit with a much more cost-effective strategy. Specifically, Hernandez et al reported a diagnostic rate of 69% in a retrospective series of 54 individuals with AVM tested with a high-depth 2000X next-generation sequencing (NGS) panel (37 genes).^17^ The estimated cost of performing such a gene panel per patient is $3000 USD, equating to a total of $162,000 USD for a cohort of 54 patients.^18^ Of the variants identified by Hernandez et al, we would have captured variants in 25 patients using our ddPCR panel providing a hypothetical diagnostic rate of 46% (25/54). We estimate a reagent cost of $110 USD per patient to complete this ddPCR screening of our targeted variants in *KRAS*, *MAP2K1*, *BRAF*, *NRAS*, and *TEK* (Supplemental Table 1), totaling ∼$5940 USD for a cohort of 54 patients. Application of the NGS panel to the remaining 29 undiagnosed patients would cost $87,000 USD, leading to the same diagnostic rate for $92,920 USD, with a cost saving of $69,060 USD. The potential utility of ddPCR screening is also highly applicable to slow-flow vascular anomalies. Specifically, Nozawa et al applied a high-depth NGS gene panel (29 genes) to achieve a diagnostic yield of 63% in 59 patients with slow-flow anomalies. Application of our ddPCR panel described here would have achieved a diagnostic yield of at least 59% within this same cohort.^16^

We demonstrate the effectiveness of ddPCR as a preliminary testing methodology achieving a high diagnostic yield for recurrent somatic variant detection in vascular anomalies or PROS. An advantage of ddPCR, is its amenability to low-quantity and quality DNA, accessible from small tissue specimens, such as shallow shave or 2 mm punch biopsies, as well as archival FFPE tissues. The fundamental principle of ddPCR relies on the partitioning of single DNA molecules into independent fluorescently labeled PCR reactions. This enables ddPCR to achieve high sensitivity for somatic variant detection with limit of detections as low as 0.25% VAF reported.^19^ The compromise of this high sensitivity is that each target variant requires a specific assay precluding novel gene or variant discovery, restricting variant identification to the variants tested and potentially limiting the detection of multiple somatic variants within one individual. Nonetheless, these limitations are negated by the genetic architecture of vascular anomalies. Specifically, common sporadic vascular malformations, where most individuals harbor a recurrent somatic variant readily amenable to detection via ddPCR, rather than a novel variant.^5^^,^^16^^,^^17^ Furthermore, although individuals with vascular anomalies harboring multiple somatic variants are certainly recognized, a recent large cohort of 936 patients with disorders of somatic mosaicism, including vascular anomalies, demonstrated only 5% of patients had multiple somatic variants.^20^ We suggest that the application of ddPCR will provide rapid genetic diagnoses, which can be performed within a single day (<4 hours). The use of ddPCR will greatly reduce the number of individuals that require costly and lengthy NGS experiments. We therefore suggest ddPCR as a cost-effective and efficacious first-line testing strategy for vascular anomalies or PROS in which variants are predominately somatic, low VAF, and recurrent, reserving NGS for those remaining undiagnosed following ddPCR.^5^^,^^16^^,^^17^

Ultimately, increasing the efficiency of pathogenic somatic variant detection in individuals with vascular anomalies will have important therapeutic implications with clinical trials of targeted treatments in the early stages for both slow-flow and fast-flow anomalies. For instance, the mTOR inhibitor Sirolimus (Rapamycin), which inhibits the PI3K-Akt pathway downstream of PI3K, has been trialed in patients with slow-flow malformations, showing the greatest efficacy in lymphatic malformations with evidence for reduced lesion volume and improved management of comorbidities, such as pain, bleeding, and quality of life.^11^ Specific inhibitors that directly target PI3K activity, such as Alpelisib, are currently being trialed in patients with PROS and confirmed *PIK3CA* variants as part of the EPIK-P2 trial having shown encouraging results in preliminary studies (https://clinicaltrials.gov/ct2/show/NCT04589650, ClinicalTrials.gov Identifier: NCT04589650).^12^^,^^13^ Furthermore, the Federal Drug Agency approved the use of Alpelisib for severe cases of PROS in 2022 (https://www.fda.gov/drugs/resources-information-approved-drugs/). Recent reports have described the efficacy of Alpelisib treatment in a cohort of 18 individuals with *TEK* or *PIK3CA*-positive VMs, demonstrating quality-of-life improvement and radiologically confirmed reduction in lesion volume.^21^ Similarly, off-label use of Alpelisib for the treatment of a patient with a *PIK3R1*-related overgrowth has also been recently demonstrated, suggesting that Alpelisib may be useful for patients with variants in related PI3K-Akt pathway genes.^14^

Targeted therapeutics are also being trialed in patients with fast-flow lesions. The compound Trametinib, which inhibits mitogen-activated protein kinase proteins encoded by *MAP2K1*, has shown promising results in 1 patient with an AVM.^15^ Accordingly, clinical trials are presently evaluating the efficacy of Trametinib in the treatment of AVMs within the prospective phase II trial, TRAMAV (https://www.clinicaltrialsregister.eu/ctr-search/trial/2019-003573-26/BE, EudraCT Number: 2019-003573-26).^22^

We demonstrate the efficacy of a tiered genetic testing strategy to provide a genetic diagnosis in 55% of our cohort. We suggest that implementation of targeted genetic screening techniques, such as ddPCR, to detect recurrent somatic variants should be considered as a rapid and cost-effective first-line investigation in individuals with vascular anomalies or PROS. Securing molecular genetic diagnoses will extend our understanding of the clinco-genetic spectrum of these disorders. Importantly, the rapid, cost-effective detection of pathogenic somatic variants may provide eligibility for current or future clinical trials of repurposed or novel targeted therapeutics.

## Data Availability

The identified variants are publicly available in the Leiden Open Variation Database.

## Conflict of Interest

The authors declare no conflicts of interest.
