# Supplementary material for: Improving genetic diagnostic yield in a large cohort of children with rare vascular anomalies or PIK3CA-related overgrowth spectrum
Source: Genet Med Open. 2023 Oct 17;2:100837. doi: 10.1016/j.gimo.2023.100837 (PMC11613910; doi:10.1016/j.gimo.2023.100837)
Supplement: Supplementary Materials [file mmc1.docx]

**Exome Sequencing Re-Analysis**

Seven individuals had clinical exome sequencing completed, the raw data from these 7 samples was obtained for re-analyses on a research basis . Briefly, reads were aligned to the hg19 reference genome with BWA-MEM v0.7.17-r1188, then duplicate marking and base quality score recalibration performed with the Genome Analysis Toolkit (GATK).^1,2^ Germline variant calling was performed with GATK HaplotypeCaller and somatic variant calling with GATK Mutect2 v4.0.1.2.^1^ Variants were annotated using vcfanno and ANNOVAR.^3,4^

We considered variants only within a curated list of 68 genes associated with vascular anomalies (**Supplementary Table 2**). Variants were further filtered according to the following criteria: located in a coding or splice site region, frequency of less than or equal to 0.001 in the Genome Aggregation Database (gnomAD v2.1.1), and variant type - missense, nonsense, coding indel or splice site.^5^

**Research Exome Sequencing**

13 individuals had exome sequencing targeting 400-fold coverage performed using the Agilent SureSelect DNA Human All Exon V6, 96RXN kit (Agilent Technologies, Santa Clara, CA, USA) and the Illumina NovaSeq 6000 System (Illumina, San Diego, CA, USA) with 150bp paired-end reads. Briefly, reads were aligned to the hg38 reference genome with BWA-MEM v0.7.17-r1188, then duplicate marking and base quality score recalibration performed with the Genome Analysis Toolkit (GATK).^1,2^ Germline variant calling was performed with GATK HaplotypeCaller and somatic variant calling with GATK Mutect2 v4.0.1.2.^1^ Variants were annotated using vcfanno and ANNOVAR.^3,4^

We considered variants only within a curated list of 68 genes associated with vascular anomalies (**Supplementary Table 2**). Variants were further filtered according to the following criteria: located in a coding or splice site region, frequency of less than or equal to 0.001 in the Genome Aggregation Database (gnomAD v2.1.1), and variant type - missense, nonsense, coding indel or splice site.^5^

| **Gene** | **ddPCR Target Variant** | **Sanger sequencing region^a^** |
| --- | --- | --- |
| ***AKT1*** | c.49G>A p.(Glu17Lys) | NT |
| ***KRAS*** | c.35G>A p.(Gly12Asp) |  |
| ***KRAS*** | c.35G>T p.(Gly12Val) |  |
| ***KRAS*** | c.35G>C p.(Gly12Ala) |  |
| ***KRAS*** | c.183A>T p.(Gln61His) |  |
| ***KRAS*** | c.34G>T p.(Gly12Cys) |  |
| ***BRAF*** | c.1799T>A p.(Val600Glu) |  |
| ***MAP2K1*** | c.167A>C p.(Gln56Pro) |  |
| ***MAP2K1*** | c.171G>T p.(Lys57Asn) |  |
| ***MAP3K3*** | c.1323C>G p.(Ile441Met) |  |
| ***TEK*** | c.2740C>T p.(Leu914Phe) | Exon 17 |
| ***TEK*** | c.2690A>G p.(Tyr897Cys) |  |
| ***TEK*** | c.2743C>T p.(Arg915Cys) |  |
| ***PIK3CA*** | c.1258T>C p.(Cys420Arg) | Exon 8  Exon 10  Exon 21 |
| ***PIK3CA*** | c.1624G>A p.(Glu542Lys) |  |
| ***PIK3CA*** | c.1633G>A p.(Glu545Lys) |  |
| ***PIK3CA*** | c.1634A>C p.(Glu545Ala) |  |
| ***PIK3CA*** | c.3140A>T p.(His1047Leu) |  |
| ***PIK3CA*** | c.3140A>G p.(His1047Arg) |  |
| ***GNA11*** | c.547C>T p.(Arg183Cys) | NT |
| ***GNA11*** | c.626A>T p.(Gln209Leu) |  |
| ***GNA11*** | c.627G>T p.(Gln209His) |  |
| ***GNAQ*** | c.548G>A p.(Arg183Gln) | Exon 5 |
| ***GNAQ*** | c.626A>C p.(Gln209Pro) |  |
| ***GNAQ*** | c.626A>T p.(Gln209Leu) |  |
| ***GNA14*** | c.614A>T p.(Gln205Leu) |  |
| ***NRAS*** | c.182A>G p.(Gln61Arg) |  |
| ***PIK3R1*** | c.1690A>G p.(Asn564Asp) |  |
| ***PIK3R1*** | c.1699A>G p.(Lys567Glu) |  |

**Supplementary Table 1.** Variants and regions targeted for ddPCR and Sanger screening. NT: not tested. RefSeq: *AKT1*: NM_001382430.1, *MAP3K3*: NM_002401.5, *PIK3CA:* NM_006218.4, *TEK*: NM_000459.5, *KRAS*: NM_004985.5, *MAP2K1*: NM_002755.4, *GNAQ*: NM_002072.5, *BRAF*: NM_004333.6, *GNA11*: NM_002067.5, *PIK3R1*: NM_181523.3, *GNA14*: NM_004297.4, *NRAS*: NM_002524.5

| *ACVRL1* | *CCM2* | *FLT4* | *GNAQ* | *KRAS* | *PDGFRB* | *SMAD4* |
| --- | --- | --- | --- | --- | --- | --- |
| *ADAMTS3* | *CCND2* | *FOXC2* | *GNB2* | *KRIT1* | *PIEZO1* | *SOS1* |
| *AGGF1* | *CDKN1C* | *GATA2* | *HGF* | *LMPH1B* | *PIK3CA* | *SOX18* |
| *AKT1* | *CDH11* | *GDF2* | *HRAS* | *MAP2K1* | *PIK3R1* | *STAMBP* |
| *AKT2* | *CELSR1* | *GJA1* | *IDH1* | *MAP3K3* | *PIK3R2* | *TEK* |
| *AKT3* | *DDX24* | *GJA4* | *IDH2* | *MET* | *PLCG1* | *TNFRSF11A* |
| *ANGPT2* | *ELMO2* | *GJC2* | *IKBKG* | *MTOR* | *PTPRB* | *TREM2* |
| *BAD* | *ENG* | *GLMN* | *IRS2* | *NF1* | *PTEN* | *VEGFC* |
| *BRAF* | *EPHB4* | *GNA11* | *KDR* | *NRAS* | *PTPN14* | *FOXO1* |
| *CCBE1* | *FAT4* | *GNA14* | *KIF11* | *PDCD10* | *RASA1* | *RIT1* |

**Supplementary Table 2.** Gene list for exome sequencing variant filtering

**References**

1. McKenna A, Hanna M, Banks E, et al. The Genome Analysis Toolkit: a MapReduce framework for analyzing next-generation DNA sequencing data. *Genome research*. 2010;20(9):1297-1303. doi:10.1101/gr.107524.110

2. Li H, Durbin R. Fast and accurate short read alignment with Burrows-Wheeler transform. *Bioinformatics*. 2009;25(14):1754-1760. doi:10.1093/bioinformatics/btp324

3. Wang K, Li M, Hakonarson H. ANNOVAR: functional annotation of genetic variants from high-throughput sequencing data. *Nucleic acids research*. 2010;38(16):e164. doi:10.1093/nar/gkq603

4. Pedersen BS, Layer RM, Quinlan AR. Vcfanno: fast, flexible annotation of genetic variants. *Genome biology*. 2016;17(1):118. doi:10.1186/s13059-016-0973-5

5. Karczewski KJ, Francioli LC, Tiao G, et al. The mutational constraint spectrum quantified from variation in 141,456 humans. *Nature*. 2020;581(7809):434-443. doi:10.1038/s41586-020-2308-7
